# Morphometric Study for Estimation and Validation of Trunk Transverse Surface Area To Assess Human Drag Force on Water

**DOI:** 10.2478/v10078-011-0017-x

**Published:** 2011-07-04

**Authors:** Jorge E Morais, Mário J Costa, Erik J Mejias, Daniel A Marinho, António J Silva, Tiago M Barbosa

**Affiliations:** 1 Polytechnic Institute of Bragança, Bragança, Portugal; 2 University of Beira Interior, Covilhã, Portugal; 3 University of Trás-os-Montes and Alto Douro, Vila Real, Portugal; 4 Research Centre in Sport, Health and Human Development, Vila Real, Portugal

**Keywords:** validation, accuracy, frontal surface area, drag, swimming

## Abstract

The aim of this study was to compute and validate estimation equations for the trunk transverse surface area (TTSA) to be used in assessing the swimmer’s drag force in both genders. One group of 133 swimmers (56 females, 77 males) was used to compute the estimation equations and another group of 131 swimmers (56 females, 75 males) was used for its validations. Swimmers were photographed in the transverse plane from above, on land, in the upright and hydrodynamic position. The TTSA was measured from the swimmer’s photo with specific software. Also measured was the height, body mass, biacromial diameter, chest sagital diameter (CSD) and the chest perimeter (CP). With the first group of swimmers, it was computed the TTSA estimation equations based on stepwise multiple regression models from the selected anthropometrical variables. For males TTSA=6.662*CP+17.019*CSD-210.708 (R^2^=0.32; R_a_^2^=0.30; P<0.01) and for females TTSA=7.002*CP+15.382*CSD-255.70 (R^2^=0.34; R_a_^2^=0.31; P<0.01). For both genders there were no significant differences between assessed and estimated mean TTSA. Coefficients of determination for the linear regression models between assessed and estimated TTSA were R^2^=0.39 for males and R^2^=0.55 for females. More than 80% of the plots were within the 95% interval confidence for the Bland-Altman analysis in both genders.

## Introduction

Swimming is considered as a human locomotion technique in the aquatic environment. Since water is not a natural environment for human beings, there is a lot of interest regarding its research. Any body, including humans, travelling in aquatic environment, is submitted to four groups of external forces: (i) weight; (ii) buoyancy; (iii) propulsive forces and; (iv) drag force.

Drag force is dependent on several hydrodynamic and anthropometrical variables including velocity, shape, size, surface area and it is similar to the general pressure drag equation (Kjendlie and Stallman, 2008):

(1) $$D=\frac{1}{2}\cdot \rho \cdot v^{2}\cdot S\cdot c_{d}$$

Where *D* is the drag force [N], ρ is the density of the water [kg.m^−3^], *v* is the swimming velocity [m.s^−1^], *S* is the projected frontal surface area of the swimmers [cm^2^] and *Cd* is the drag coefficient (changing according to shape, orientation and Reynolds number).

The assessment of the drag force can be done with the swimmers towing in water and without segmental actions (i.e. passive drag) while the subject is making segmental actions to propeller him/herself (i.e. active drag) (Pendergast et al., 2006; Marinho et al., 2009). Passive and active drag can be measured with numerical simulations or experimental methods. Numerical solutions use techniques such as computer fluid dynamics (CFD) (e.g. Silva et al., 2008; Marinho et al., 2010a). For a passive drag measurement, there are some methods reported in the literature (e.g. Clarys et al., 1974; Zamparo et al., 2009). Subjects are passively towed on prone and hydrodynamic position holding a wire in the hands. An engine roll up the wire at a constant speed and the resistance force is measured by a dynamometer. On the other hand, for active drag, with more citations in the literature, there is a method describing the interpolation of oxygen uptake for null drag when swimming with extra positive and negative loads (di Prampero et al., 1974), the measuring active drag-system apparatus (Hollander et al., 1986; Toussaint et al., 2004) and the velocity perturbation method (VPM) (Kolmogorov and Duplischeva, 1992; Kolmogorov et al., 2000).

Anthropometrics, such as body size or body density (Zamparo et al., 1996) has a significant influence on drag force. A couple of methods to assess drag force (i.e. *CFD* and *VPM* methods) need to include in the data input the trunk transverse surface area (TTSA). The *TTSA* on regular basis is also called by practitioners and researchers of “frontal surface area” or “projected surface area on the direction of displacement” or even “body cross-sectional diameter”. The *TTSA* can be directly measured in each subject and inserted in the data input of the *CFD* and *VPM* methods. TTSA is measured with a planimeter, on screen measure area software of plane 2D digital images, or body scan (Nicolas et al., 2007; Nicolas and Bideau, 2009). However, TTSA data collection and its treatment are somewhat time consuming and/or expensive. Therefore, most of the times practitioners and researchers estimate *TTSA* based on some selected anthropometrical variables. Clarys (1979) suggested a *TTSA* estimation equation based on the subject’s body mass and height (R^2^ = 0.50):

(2) TTSA=6.9256⋅BM+3.5043⋅H−377.156

Where *TTSA* is the trunk transverse surface area [cm^2^], *BM* is the body mass [kg] and *H* is the height [cm].

This estimation equation was developed using stepwise regression models that included several anthropometrical variables of 63 physical education students and 9 Olympic swimmers. Equation 2 is on regular basis used to assess drag force in children (Kjendlie and Stallman, 2008; Marinho et al., 2010b; Barbosa et al., 2010c) and adult swimmers (Kolmogorov and Duplischeva, 1992), male and female subjects (Kolmogorv et al., 2000; Toussaint et al., 2004) without a clear knowledge of the good-of-fit of the model to different cohort groups. Moreover, the research was performed in the seventies. Anthropometrical characteristics of the 70’s swimmers are not the same as the ones of the XXI century.

The aim of this study was to compute and validate *TTSA* estimation equations to assess the swimmer’s drag force in both genders. It was hypothesized that it is possible to compute accurate and valid equations to estimate *TTSA* for male and female swimmers in a broad range of ages.

## Material and methods

### Sample

Total sample was composed of 264 subjects (152 males and 112 females). All subjects were competitive swimmers with regular participation in competitions at the regional, national, or international level. Swimmers chronological ages ranged between 10–32 years old for males and 9–27 years old for females.

**Figure 1:**
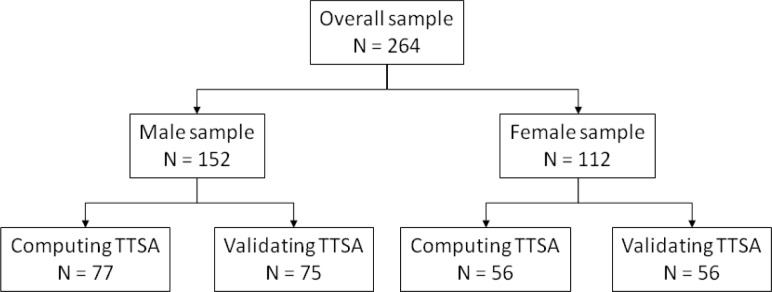
The split of overall sample to compute and validate the trunk transverse surface area (TTSA).

Total sample was divided into two groups based on gender. In each gender group the sub-sample was divided once again: (i) approximately half of subjects were used to compute the *TTSA* estimation equations and; (ii) the other half for its validation. One group of 133 swimmers (56 females and 77 males) was used to compute the TTSA estimation equations and another group of 131 swimmers (56 females and 75 males) was used for its validations. Figure 1 presents the split of the sample.

All procedures were in accordance to the Declaration of Helsinki in respect to Human research. The Institutional Review Board of the Polytechnic Institute of Bragança approved the study design. Subjects (or when appropriate their legal tutors) were informed of the potential experimental risks and signed an informed consent document prior to data collection.

### Data Collection

For the *TTSA* measurement, subjects were photographed with a digital camera (DSC-T7, Sony, Tokyo, Japan) in the transverse plane from above (Caspersen et al., 2010). Subjects were on land, in the upright and hydrodynamic position. This position is characterized by the arms being fully extended above the head, one hand above the other, fingers also extended close together and head in neutral position. Subjects wore a regular textile swimsuit, a cap and goggles. Besides the subjects, on the camera shooting field there was a calibration frame with 0.945 [m] length at the height of the xiphoid process. *TTSA* was measured from the subject’s digital photo with a specific software (Udruler, AVPSoft, USA). Procedures included: (i) scale calibration; (ii) manual digitalization of the transverse trunk perimeter; (iii) output and recording of the *TTSA* value.

Also measured were the following selected anthropometrical variables: (i) body mass; (ii) height; (iii) biacromial diameter; (iv) chest sagital diameter and; (v) chest perimeter. Most of these variables are reported on regular basis in competitive swimming anthropometrical reports and research papers (e.g. Mazza et al., 1994). All measurements were carried-out wearing a regular textile swimsuit, a cap and goggles. Body mass (BM) was measured in the upright position with a digital scale (SECA, 884, Hamburg, Germany). Body height (H) was measured in the anthropometrical position from vertex to the floor with a digital stadiometer (SECA, 242, Hamburg, Germany). Biacromial diameter (BCD) is considered as the distance between the two acromion processes. Chest sagital diameter (CSD) is considered as the distance between the back and the highest point of the chest (i.e. antero-posterior) at the level of the xiphoid process. Both diameters were measured once again with a specific sliding calliper (Campbell, 20, RossCraft, Canada) being the subjects in the anthropometrical position. Chest perimeter (CP), defined as the perimeter of the trunk at the level of the xiphoid process, was measured with a flexible anthropometrical tape (RossCraft, Canada). All anthropometrical evaluations were performed by an expert. Each anthropometrical variable was measured three consecutive times. For further analyses, the mean value of all three trials was considered.

### Statistical procedures

The normality and homocedasticity assumptions were checked respectively with the Kolmogorov-Smirnov and the Levene tests. Descriptive statistics (mean, one standard deviation, minimum, maximum and coefficient of variation) from all measured variables were calculated.

In the first sub-sample group forward step-by-step multiple regression models were computed. *TTSA* was considered as endogenous variable and remaining anthropometrical variables (i.e. body mass, body height, *BCD*, *CSD* and *CP*) as exogenous variables. The variables entered the equation if F≥ 4.0 and removed if F≤ 3.96 as suggested elsewhere (Barbosa et al., 2008). All assumptions to perform the selected multiple regression models were taken into account. For further analyses the equation computed, the coefficient of determination (R^2^), the adjusted coefficient of determination (Ra^2^), the error of estimation (s) and the probability of rejecting the null hypothesis (p ≤ 0.05). In each exogenous variables included in the final model, the t-value and the p-value were considered as well.

Validation was made in the second sub-sample group (Baldari et al., 2009; Kristensen et al., 2009; Wolfram et al., 2010): (i) comparing mean data; (ii) computing simple linear regression models and; (iii) computing Bland Altman plots. Comparison between the mean TTSA assessed and the *TTSA* estimated, according to the equations previously developed, was made using paired Student’s t-test (p ≤ 0.05). Simple linear regression model between both assessed and estimated *TTSA* was computed. As a rule of thumb, for qualitative and effect size analysis, it was defined that the relationship was: (i) very weak if R^2^ < 0.04; weak if 0.04 ≤ R^2^ < 0.16; moderate if 0.16 ≤ R^2^ < 0.49; high if 0.49 ≤ R^2^ < 0.81 and; very high of 0.81 ≤ R^2^ < 1.0. In addition, the error of estimation (s) and the confidence interval for 95 % of the adjustment line in the scatter gram was computed. The Bland Altman analysis (Bland and Altman, 1986) included the plot of the mean value of *TTSA* assessed and estimated versus the delta value (i.e. difference) between *TTSA* assessed and estimated. It was adopted as limits of agreement a bias of ± 1.96 standard deviation of the difference (average difference ± 1.96 standard deviation of the difference). For qualitative assessment, it was considered that *TTSA* estimated was valid and appropriate if at least 80% of the plots were within the ± 1.96 standard deviation of the difference.

## Results

### Morphometric characteristics

Table 1 presents the descriptive statistics for all selected anthropometrical variables, according to gender groups. Overall, it can be verified that most mean values are higher in male than in female subjects. Data dispersion can be considered as weak (i.e. CV ≤ 15%) or moderate (i.e. 15% < CV ≤ 30%) within each gender group.

### Computation of trunk transverse surface area prediction models

For male gender, the final model (F_2.75_ = 17.143; p < 0.001) included the *CP* (t = 2.963; p < 0.001) and the *CSD* (t = 2.333; p = 0.02) in order to predict the *TTSA*. The equation was (R^2^ = 0.32; Ra^2^ = 0.30; s = 158.93; p < 0.01):

(3) TTSA=6.662⋅CP+17.019⋅CSD−210.708

For the female gender, the final model (F_2.53_ = −12.871. p < 0.001) included the *CP* (t = 3.760; p < 0.001) as well as the *CSD* (t = 2.837; p = 0.01). The *TTSA* estimation equation was (R^2^ = 0.34; Ra^2^ = 0.31; s = 119.22; p < 0.01):

(4) TTSA=7.002⋅CP+15.382⋅CSD−255.70

### Validation of trunk transverse surface area prediction models

**Figure 2:**
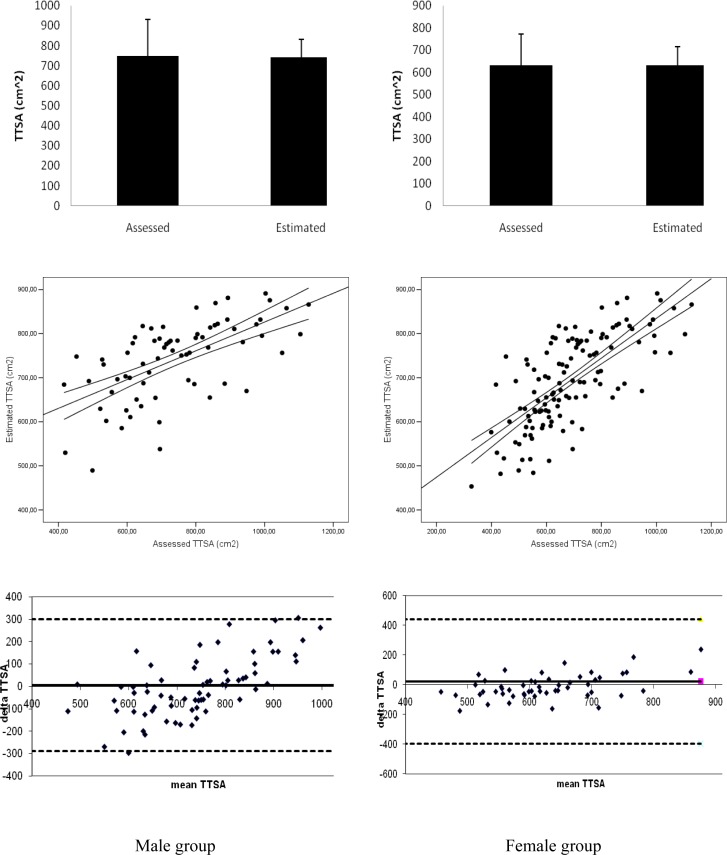
Comparison of mean data, scatter gram and Bland Altman plots between assessed and estimated trunk transverse surface areas (TTSA).

Figure 2 presents the comparison of mean data, scatter gram and Bland Altman plots between assessed and estimated *TTSA* based on equations 3 and 4, for male and female genders, respectively. For male subjects, mean value of assessed TTSA was 747.27 ± 182.38 [cm^2^] and the estimated one was 741.54 ± 89.02 [cm^2^]. In female subjects, mean *TTSA* data assessed was 630.25 ± 142.14 [cm^2^] and the estimated FSA was 631.57 ± 83.04 [cm^2^]. Comparing assessed and estimated TTSA, mean data was non-significant (p > 0.05).

The scatter gram analysis for male (R^2^ = 0.39; s = 70.14; p < 0.001) and female (R^2^ = 0.55; s = 71.68; p < 0.001) genders revealed statistically significant coefficients of determination ranging from moderate to high relationships.

For the Bland Altman plots, in the female group, none dot was located beyond the 1.96 SD limits. In the male plots, only two dots were beyond the agreement limits. So, the cut-off value of at least 80% of the plots within the ± 1.96 SD was accomplished for male and female groups.

## Discussion

The aim of this study was to compute and validate estimation equations for the trunk transverse surface area in order to be used to assess the swimmer’s drag force in both genders. The computed *TTSA* equations based on the *CP* and *CSD* can be considered as valid to assess drag force in both genders in a broad range of ages from children to young adults.

### Morphometric characteristics

In order to compute and validate *TTSA* estimation equations, a somewhat high sample size was selected. Previous research reported that some anthropometrical variables are related to *TTSA*. Clarys (1979) verified that the height and body mass were the exogenous variables able to predict *TTSA* with a higher coefficient of determination. Huijing et al. (1988) observed significant relationships between *TTSA* and several other variables besides height and body mass in 17 male swimmers. Indeed, in the mentioned paper, the variables with significant association level to *TTSA* were the estimated body surface, all measured segmental circumference, arm’s and leg’s lengths. However, authors did not report significant associations with most of the distances, such as *BCD* and thorax depths. This lack of significant association might be related to the reduce of data statistical power, since a small and homogeneous sample size was used. *TTSA* from a geometrical point of view is quite similar to a circle or an oval shape. Geometrically, a circle area is computed as:

(5) $$A_{c}=\cdot \pi \cdot r^{2}$$

Where *A_c_* is the circle area [m^2^], π a constant value of 3.14 and *r* is the radius [m].The area of an oval or ellipse is found:

(6) $$A_{o}=w\cdot l\cdot 0.8$$

Where *A_o_* is the oval area [m^2^], *w* is the width [m] and *l* the length [m]. So, transferring the geometrical knowledge to anthropometrics, it seems that the breaths are the exogenous variables that might be able to predict more powerful *TTSA* estimation equations. Added to this we had approximately 75 male and 55 female subjects to compute and additional ones to validate the estimation equations using forward step-by-step multiple regression models. When computing multiple regression models it is stated that it is necessary to consider at least 15 subjects for each exogenous variables inserted in the model (i.e. K > 15). Therefore, our decision was to insert 5 exogenous variables (i.e. body mass, height, BCD, CSD and CP) trying to maintain some data consistence. Body mass and height were inserted because they are the variables used in equation 2. The *BCD*, *CSD* and *CP* were added because geometrically they seem to be the variables that allow a higher *TTSA* estimation.

Analyzing the descriptive data presented in Table 1, mean values are similar or slightly lower than other papers reporting anthropometrical data (Mazza et al., 1994; Strzała et al., 2005; 2007; Knechtke et al., 2010) and TTSA (Nicolas et al. 2007; Nicolas and Bideau, 2009; Caspersen et al., 2010). This research presents a higher dispersion data, as the age range is also higher. Remaining papers focused on stricter chronological age frames or even made separate groups analysis for children and adults. In this sense, it can be speculated that data is in accordance with the main literature. The development of biomechanical models, in this case a statistical one estimating the *TTSA* based on selected anthropometrical variables, can be a feasible way to promote hydrodynamic evaluation (i.e. drag force) with relevant information for swimmers and coaches (Barbosa et al., 2010a). So, being descriptive statistics similar to main literature and presenting moderate dispersions it allowed to compute and validate the biomechanical models (Barbosa et al., 2010b), as in this case the *TTSA* estimation equations, based on these data.

### Computation of trunk transverse surface area prediction models

For both male and female gender the final model for the *TTSA* estimation equations included the *CP* and the *CSD*. The equations were significant and with a prediction level qualitatively considered as moderate. This means that some other variables not considered for the prediction can have some impact on the *TTSA* estimation. Forcing new variables entering the model could increase slightly the coefficient of determination but, would also increase the error of estimation. In this sense, it was decided to maintain the true nature of the model developed and not forcing other variables to be included on it.

Equations 3 and 4 have a coefficient of determination lower than equation 2. The explanation for that might be our decision to compute estimation equations for a broad range of ages and not only for young adults. Added to that, unfortunately, the procedures used to validate equation 2 are not known as it was reported in a review paper instead of an original research type. Moreover, such equation was developed for male swimmers but it is often used for female ones and even with children of both genders because, for the best of our knowledge, there is no other one computed and validated for those groups. That is the reason why we attempted to develop equation models that are fitted and validated not only for male, but also for female swimmers and children of both genders.

From a mathematical point of view (i.e. geometrics), we speculated that other anthropometrical variables besides height and body mass could have higher prediction ability. Indeed, the models to compute equations 3 and 4 excluded body mass and height, inserting some length variables (i.e., *CP* and *CSD*). At last, it can be stated that the prediction error can be considered as reduced, especially for the female gender.

### Validation of trunk transverse surface area prediction models

After developing a new apparatus, technical or methodological procedure it is wise to validate it. On a regular basis the validation process included: (i) the comparison of the mean values between a gold standard and the new procedure; (ii) establishment of the relationship between the gold standard and the new procedure and; (iii) assessment if the difference between the measurements by the two methods is related to the magnitude of the measurement. Several authors considered that some of these procedures are inappropriate for such an aim. Bland and Altman (1986) do not agree with the use of the correlation/determination coefficients. On the other hand, Hopkins (2004) considered that the Bland Altman plot of difference versus mean values for the method and criterion shows a systematic proportional bias in the method’s readings, even though none is present, which do not happens on a regression analysis of the criterion versus the instrument shows no bias. It must be stressed that our paper is not about validation techniques. Because there is no consensual opinion, on a regular basis, the three procedures are used on several of fields knowledge such as Physiology (Baldari et al., 2009), Motor Control and Posture (Kristensen et al., 2009), Anthropometrics (Siahkouhian and Hedayatneja, 2010) or Biomechanics (Wolfram et al., 2010).

There were no significant differences between measured *TTSA* and estimated *TTSA*. The coefficients of determination between both variables were significant. Added to that, any Bland Altman analysis presented less than 80% of the plots within the ± 1.96 SD. So, all procedures suggest that equations 3 and 4 are valid ways to assess *TTSA* on male and female genders, respectively. Validations were carried-out with groups of subjects with similar characteristics of the ones used to compute *TTSA*. So, validation is only considered for same range of ages and gender. It is questionable if equations 3 and 4 are suitable to be used in other subjects.

It can be considered as main limitations of this original research: (i) *TTSA* computed are only appropriate for subjects from children (i.e. approximately 6 years-old) to young adults (approximately 30 years-old) and not being valid for remaining ages; (ii) computed equations are not sensitive to the subjects sports level; (iii) adding or forcing extra anthropometrical variables to enter in the final model, it might increase the *TTSA* estimation level, but data collection will become more time consuming.

As a conclusion: (i) both *TTSA* estimation models computed were significant and with moderate coefficients of determination; (ii) between mean values of assessed and estimated, TTSA was not significantly different; (iii) coefficients of determination between assessed versus estimated *TTSA* ranged between moderate and high relationships and; (iv) cut-off values adopted for the Bland Altman Plots were accomplished. In this sense, it can be stated that the models developed can be used with validity to estimate *TTSA* for both male and female subjects in a broad range of ages, from children to young adults.

## Figures and Tables

**Table 1:** Anthropometrical characteristics of male (M) and female (F) subjects for body mass (BM), body height (H), biacromial diameter (BCD), chest sagital diameter (CSD), chest perimeter (CP) and measured trunk transverse surface area (TTSA)

	BM [kg]		H [cm]		BCD [cm]		CSD [cm]		CP [cm]		TTSA [cm^2^]	

	M	F	M	F	M	F	M	F	M	F	M	F
Mean	63.61	50.04	169.41	157.46	35.41	33.13	22.43	21.57	86.90	78.08	747.46	634.23
1 SD	15.10	10.04	12.12	9.37	5.07	4.85	3.00	2.85	9.31	8.41	184.59	144.56
Minimum	28.00	27.80	134.00	133.00	19.90	24.20	11.50	15.50	61.50	64.00	373.59	327.21
Maximum	108.60	72.20	189.00	178.00	50.50	44.00	31.00	28.10	112.00	97.00	1371.00	1125.20
CV	23.74	20.06	7.15	5.95	14.32	14.64	13.37	13.21	10.71	10.77	24.70	22.79
